# Prevalence of hyperuricemia in patients with severe obesity and the relationship between serum uric acid and severe obesity: A decade retrospective cross-section study in Chinese adults

**DOI:** 10.3389/fpubh.2022.986954

**Published:** 2022-08-26

**Authors:** Chonin Cheang, Saikam Law, Jieru Ren, Wengtong Chan, Cunchuan Wang, Zhiyong Dong

**Affiliations:** ^1^School of Medicine, Jinan University, Guangzhou, China; ^2^Department of Bariatric Surgery, The First Affiliated Hospital of Jinan University, Guangzhou, China

**Keywords:** hyperuricemia, obesity, serum uric acid, Chinese, retrospective cross-sectional study

## Abstract

**Objective:**

Undoubtedly, the relationship between serum uric acid (SUA) and obesity is less data for Chinese patients with obesity. This study aimed to examine the prevalence of hyperuricemia (HUA) and the association between SUA and patients with obesity.

**Methods:**

All participants were categorized as overweight, obesity I, obesity II, and obesity III. In addition, based on SUA concentration, the participants were stratified into four quartiles. The authors used descriptive analysis, independent *t*-test, ANOVA, correlation analysis, and multiple linear regression models to verify the SUA level and obesity among Chinese adults.

**Results:**

Overall, the estimated prevalence of HUA was 69.8%. In the BMI categories, the prevalence of HUA was 5.1% in overweight, 15.2% in obesity I, 16.9% in obesity II, and 32.5% in obesity III. Correlation analysis shows that SUA is strongly correlated with BMI, waist circumference (WC), and hip circumference (HC). Multiple linear regression analysis shows that high density–lipoprotein cholesterol (HDL-C) is a protective predictor of serum uric acid levels in patients with obesity. Compared with the overweight, obesity I, obesity II, and obesity III were more likely to have higher levels in the SUA levels.

**Conclusion:**

We mainly showed that the serum uric acid levels in Chinese patients with severe obesity declined slightly as age increased.

## Introduction

Obesity is an increasingly severe clinical and public health problem, with more than 100 million patients with obesity worldwide (1). Obesity has emerged as a major public health concern in China. Based on criteria for the Chinese population, more than half of Chinese adults had either overweight or obese in the most recent national survey. Overweight and obesity incidence was 34.3 and 16.4% (2). Insulin resistance, type 2 diabetes, hypertension, and hyperuricemia (HUA) are among endocrine disorders that can be caused by obesity and metabolic syndrome (3, 4), which places a heavy burden on patients, families, and the public health system.

In addition, Serum uric acid (SUA) is the end-product of purine metabolism in humans (5). Approximately two-thirds of SUA is produced endogenously, and the remaining is due to abundant diet purines (6). One of the key reasons for HUA and gout development is abnormal SUA metabolism and reduced excretion by the kidneys (7, 8). Elevated SUA can lead to gout and is an essential risk factor for obesity, resulting in increased metabolic syndrome (9, 10). Several studies have confirmed that patients with obesity, especially those with abdominal obesity, are independent predictors of HUA development (11, 12). A cross-sectional study showed that body mass index (BMI) significantly increases with elevated SUA among 27,009 middle-aged and elderly Chinese adults (13); another research still supports that (14).

In China, the prevalence of HUA was 13.3%, male and female was 19.4 and 7.9% (8). SUA was positively associated with numerous indices, including BMI, waist circumference, and dyslipidemia, in epidemiological investigations on metabolic syndrome (15, 16). A substantial positive relationship between SUA and obesity in Chinese adult population has been discovered (9, 17), Japan (18), India (15), Pakistan (16), and Bangladeshi (14). Accordingly, the present study was carried out to analyze the prevalence of HUA in Chinese obese/overweight adults and assessed the relationship between SUA and obesity by analyzing the metabolic status and related influencing factors of patients with obesity (even severely obese).

## Research methodology

### Study design and subject

This study was a retrospective cross-sectional design conducted between January 2010 and December 2020. Subjects comprised 821 patients with obesity (304 males and 517 females) admitted to the author's hospital. Our analysis looked retrospectively at a large cohort of patients before being treated. All procedures were performed following ethical standards. This study have been approved by the hospital of the ethics committee. Inclusion criteria were BMI ≥ 25 kg/m^2^ and Chinese citizen. Exclusion criteria were severe hepatic and renal insufficiency, severe cardiovascular and cerebrovascular diseases, serious psychiatric illness, alcohol or drug abuse, taking Chinese traditional medicine drugs, uric acid–lowering drugs, angiotensin-receptor blockers, or diuretics within 3 months before admission, and high fat and high purine diet within 1 month before admission, and pregnant or breastfeeding.

### Measurements

Trained health technicians used a structured questionnaire form to record the baseline anthropometric measurements. Individual height, weight, waist circumference (WC; measured at the level of the umbilicus), and hip circumference (HC; measured as the horizontal circumference at the most prominent area of the hips), as well as other lifestyle variables, were gathered following the standard approach described elsewhere (19). Every day, the scales were calibrated to a standard. The body mass index (BMI) was determined by dividing the weight (in kg) by the height (in meters; in m^2^). Repeated measurements in the presence of investigators proved the accuracy of the anthropometric data. Laboratory measurements were obtained to measure SUA, total cholesterol (TC), low density–lipoprotein cholesterol (LDL-C), and high density–lipoprotein cholesterol (HDL-C) following a fast of 8–12 h, and venous blood was collected from each patient. Blood lipids and SUA were measured by immunoturbidimetry.

### Outcomes and definitions

In present study, SUA concentrations of > 7.0 mg/dL (416.4 mol/L) in men and > 6.0 mg/dL (356.9 mol/L) in women were used to identify HUA (8, 20).These cutoff values were chosen because they are often utilized in clinical laboratories and have been advocated in prior studies for determining HUA (20). SUA levels were categorized into four quartiles to compare the prevalence of obesity and its association with SUA quartiles. Based on diagnostic criteria of obesity for Chinese populations recommended by the WHO, we categorized BMI into four groups: overweight (≥25–29.9 kg/m^2^), obesity I (30–34.9 kg/m^2^), obesity II (35–39.9 kg/m^2^), and obesity III (≥40 kg/m^2^) (21). WC ≥ 90 cm for men and ≥ 80 cm for women was used to determine abdominal obesity (22). So, the present study used SUA levels categorized into (HUA and non-HUA) to compare the prevalence of each obesity category and the relationship between SUA four quartiles and each obesity category.

### Statistical analysis

IBM SPSS Statistics version 26 (IBM SPSS Statistics, New York, United States) was used to analyze all of the data. The differences between male and female participants for anthropometric and baseline factors were assessed using a two-tailed independent sample *t*-test. Pearson's correlation coefficient test was used to analyze the interrelationships between anthropometric, baseline factors, and SUA. To determine differences between the groups, a one-way ANOVA was used. The degree of SUA, and the factors that influence obesity were studied using multiple linear logistic regression. Unless otherwise stated, the values in the tables were given as mean and standard deviation (SD). For statistical significance, a level of alpha 0.05 was chosen.

## Results

### Baseline characteristics of the study

The basic characteristics of the study are summarized in Table 1. Of the 821 subjects, 37.0% were males, and 63.0% were females. The mean age of the participants was 31.9 ± 9.3 years (range 18–71 years). The average BMI for all subjects was 39.0 ± 8.5 kg/m^2^, with a significant difference between males and females (*p* < 0.001). The mean value of WC and HC were 120.5 ± 18.0 and 123.8 ± 15.6, with a significant difference between male and female (*p* < 0.001) subjects. Notably, all participants were abdominal obesity (WC ≥ 90 cm for men and ≥ 80 cm for females). Among them, the male was observed for the mean level of SUA (*p* < 0.001), and TG (*p* < 0.01) was significantly higher than the female. In the contract, females' HDL levels are higher than for males (*p* < 0.001).

**Table 1:** Baseline characteristics and SUA level of the study cohort.

**Variable**	**Total**	**Male**	**Female**	***P*** **-value**
Number (n)	821	304	517	-
Age (years)	31.9 ± 9.4 (71)	31.5 ± 9.9 (71)	32.1 ± 9.1 (68)	0.117
Height (cm)	166.1 ± 8.6 (191.1)	173.5 ± 7.0 (191.1)	161.8 ± 6.1 (186.6)	<0.001
Weight (kg)	108.6 ± 28.8 (249.1)	128.6 ± 30.3 (249.1)	96.8 ± 20.0 (215.5)	<0.001
WC (cm)	120.5 ± 18.0 (186)	131.9 ± 18.0 (184.0)	113.8 ± 14.2 (186.0)	<0.001
HC (cm)	123.76 ± 15.6 (200.0)	128.8 ± 17.2 (200.0)	120.8 ± 13.7 (181.5)	<0.001
BMI (kg/m^2^)	39.0 ± 8.5 (97.1)	42.5 ± 9.4 (97.1)	36.9 ± 7.2 (80.0)	<0.001
SUA (μmol/L)	442.5 ± 122.8 (949.4)	502.5 ± 130.0 (949.4)	407.2 ± 103.4 (809.2)	<0.001
TG (mg/dl)	180 ± 146.0 (1,488.5)	224.6 ± 193.9 (1,488.5)	153.7 ± 99.7 (953.3)	<0.001
TC (mg/dl)	193.8 ± 39.4 (440.4)	195.7 ± 44.6 (440.4)	192.7 ± 35.9 (353.7)	0.271
HDL-C (mg/dl)	40.1 ± 15.6 (367.7)	37.7 ± 12.5 (142.0)	41.5 ± 17.0 (367.7)	<0.001
LDL-C (mg/dl)	118.7 ± 50.6 (1,298.4)	117.8 ± 30.7 (226.4)	119.3 ± 59.3 (1,298.4)	0.427

Data are presented as mean ± SD. Values in parentheses indicate maximum level of the parameter. P-value obtained from independent sample t-test in comparison the gender group.

BMI, body mass index; HC, hip circumference; HDL, high-density lipoprotein cholesterol; LDL, low-density lipoprotein cholesterol; WC, waist circumference; SUA, serum uric acid; TC, total cholesterol; TG, triglycerides.

### Prevalence of HUA among the patients with obesity

Based on the diagnostic criteria, 573 participants were identified as HUA individuals. Overall, the estimated prevalence of HUA was 69.8%, with 77.0% in males and 65.6% in female participants (Table 2). In the BMI categories, the prevalence of HUA was 5.1% in overweight, 15.2% in obesity I, 16.9% in obesity II, and 32.5% in obesity III. The mean level of SUA was 313.1 ± 47.2 μmol/L (max 414.9 μmol/L) and 498.5 ± 101.3 μmol/L (max 949.4 μmol/L) in the non-HUA and HUA group, respectively. A significant difference was observed for SUA, age, BMI, WC, and HC between non-HUA and HUA groups (*p* < 0.001, Table 2).

**Table 2:** Comparison of baseline characteristics between non-hyperuricemia and hyperuricemia subjects.

**Variable**	**Non-hyperuricemia (*n* = 248)**	**Hyperuricemia (*n* = 573)**	***P*** **-value**
Male (*n* = 304)	70 (34.4%)	234 (77.0%)	-
SUA (μmol/L)	335.1 ± 55.0 (414.9)	552.6 ± 100.6 (949.4)	<0.001
Female (*n* = 517)	178 (34.4%)	339 (65.6%)	-
SUA (μmol/L)	304.4 ± 40.7 (356.0)	461.1 ± 83.5 (809.2)	<0.001
Age (years)	34.7± 9.8	30.7 ± 9.0	<0.001
BMI (kg/m^2^)	35.9 ± 7.2	40.4 ± 8.7	<0.001
WC (cm)	113.8 ± 15.9	123.4 ± 18.1	<0.001
HC (cm)	118.2 ± 13.2	126.1 ± 15.9	<0.001
TG (mg/dl)	175.0 ± 166.8	182.2 ± 136.1	0.516
TC (mg/dl)	190.9 ± 38.6	195.1 ± 39.6	0.155
HDL-C (mg/dl)	41.1 ± 11.4	39.6 ± 17.1	0.219
LDL-C (mg/dl)	116.7 ± 80.1	119.6 ± 30.0	0.461

Values are presented as mean ± SD. SUA level indicated in parentheses as the maximum. Hyperuricemia was defined as the SUA level in men> 416.4 (7 mg/dl) and in women >356.9 (6 mg/dl) by Sui et al.

BMI, body mass index; HC, hip circumference; HDL, high-density lipoprotein cholesterol; LDL, low-density lipoprotein cholesterol; WC, waist circumference; SUA, serum uric acid; TC, total cholesterol; TG, triglycerides.

### Correlation between of SUA and risk factors of obesity

**Figure 1:**
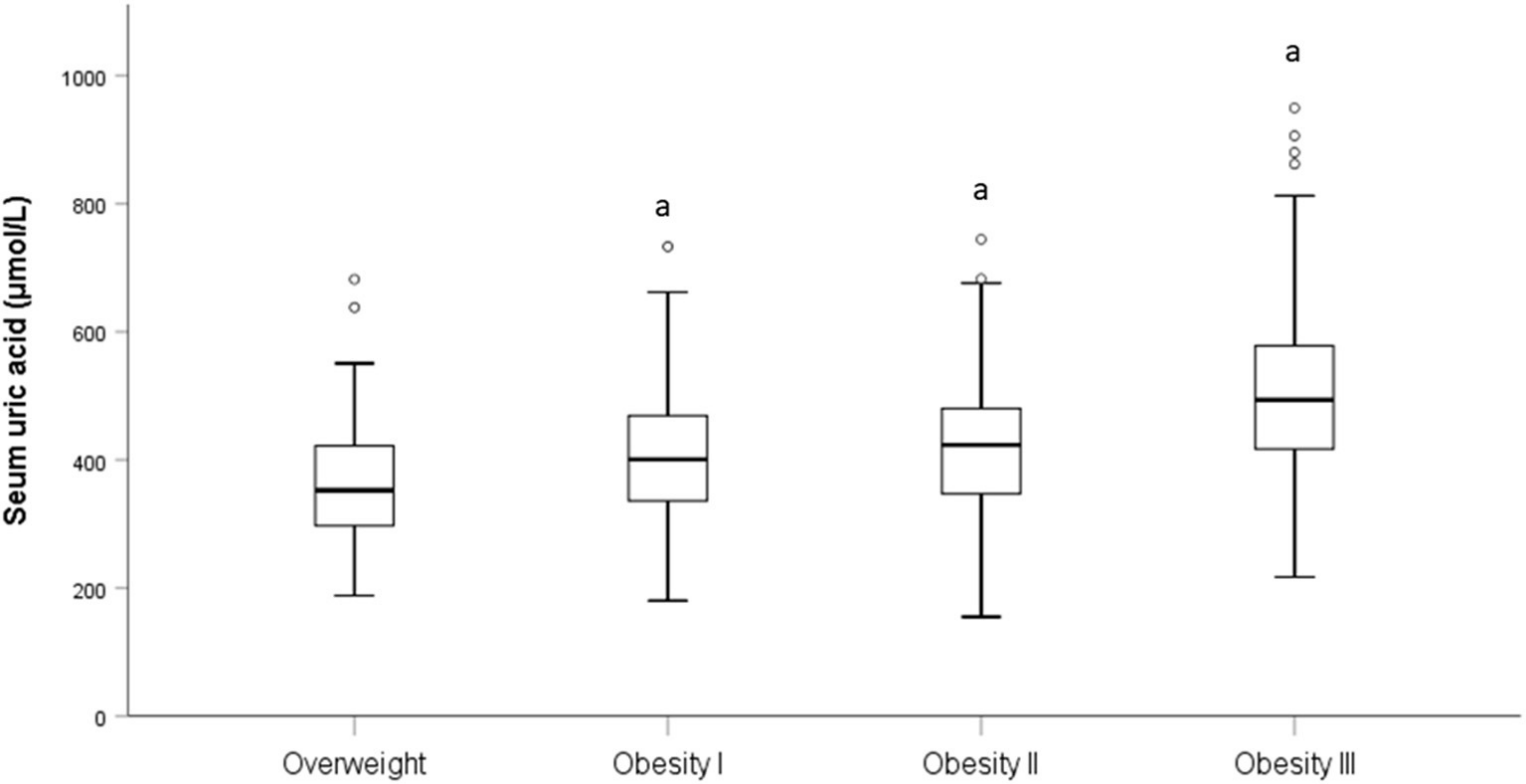
Level of SUA in different BMI category. “a” is represent *p* < 0.001 when compared to the overweight category.

Table 3 presents the correlation of SUA with all subjects. The correlation analysis demonstrated the strong positive correlation of SUA with BMI (*p* < 0.001), WC (*p* < 0.001), and HC (*p* < 0.001); low negative correlation of SUA with age (*p* < 0.001). After adjusted age and sex, SUA still demonstrated a strong correlation between BMI, WC, and HC (*p* < 0.001). When BMI was categorized into overweight, obesity I, obesity II, and obesity III groups, a significant difference in SUA levels were found in obesity I (*p* < 0.001), obesity II (*p* < 0.001), and obesity III (*p* < 0.001) group when compared to overweight group (Figure 1).

**Table 3:** Correlation coefficients between SUA and all parameters.

**Variable**	**No calibration**		**Calibration age, sex**	
	***R***	***P***	***R***	***P***
Age	−0.222	<0.001	-	-
BMI (kg/m^2^)	0.352	<0.001	0.246	<0.001
TGs (mmol/L)	0.096	0.006	0.023	0.511
TC (mmol/L)	0.146	<0.001	0.149	<0.001
HDL-C (mmol/L)	−0.099	0.004	−0.051	0.147
LDL-C (mmol/L)	0.097	0.005	0.116	0.001
WC	0.410	<0.001	0.272	<0.001
HC	0.327	<0.001	0.240	<0.001

BMI, body mass index; HC, hip circumference; HDL, high-density lipoprotein cholesterol; LDL, low-density lipoprotein cholesterol; WC, waist circumference; SUA, serum uric acid; TC, total cholesterol; TG, triglycerides.

### SUA quartiles and comparison of obesity between the quartiles

**Figure 2:**
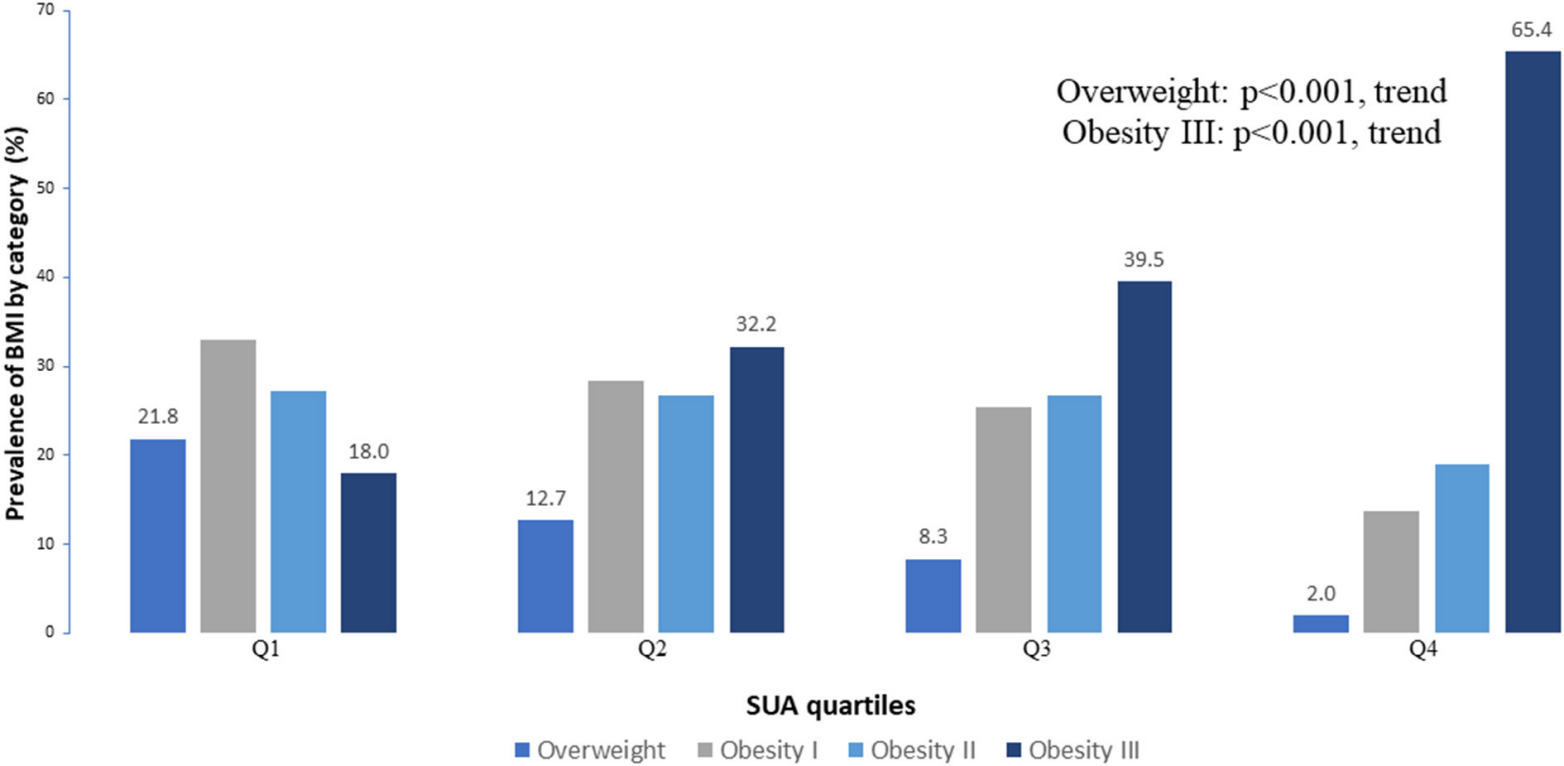
Comparison of obesity category between the SUA quartile groups.

Table 4 shows the baseline characteristics of the individuals according to SUA quartiles. As expected, the participants with higher SUA quartiles were more likely to be men. Across the SUA quartiles, BMI, WC, HC, SUA, TG, TC, and LDL levels steadily increased, while HDL levels progressively declined (Table 4). The prevalence of overweight was significantly decreased with the increasing SUA quartile (21.8, 12.7, 8.3, and 2.0% for the first, second, third, and fourth quartiles, respectively, *p* < 0.001 for trend). In contrast, the prevalence of obesity III was significantly increased with the increasing SUA quartile (18.0, 32.2, 39.5, and 65.4% for the first, second, third, and fourth quartiles, respectively, *p* < 0.001 for trend; Table 5 and Figure 2).

**Table 4:** Characteristics of the subjects according to SUA (μmol/L) quartiles.

	**Q1**	**Q2**	**Q3**	**Q4**		
**Variable**	**<351**	**351–429.4**	**429.401–515.5**	**>515.5**	***F***	***P*** **-value**
Number (*n*)	206	205	205	205	-	-
Gender (m/f)	40/166	39/166	91/114	134/71	-	
Age (years)	35.0 ± 9.9	32.2 ± 9.0	30.9 ± 8.9	29.4 ± 9.0	13.59	<0.001
WC (cm)	112.4 ± 15.4	116.2 ± 15.5	121.3 ± 15.9	132.1 ± 18.7	56.01	<0.001
HC (cm)	117.7 ± 13.4	112.1 ± 14.0	123.6 ± 14.3	131.7 ± 17.0	32.54	<0.001
BMI (kg/m^2^)	35.5 ± 7.3	37.8 ± 7.5	39.0 ± 7.4	43.8 ± 9.5	39.05	<0.001
SUA (μmol/L)	300.1 ± 39.7	392.9 ± 25.3	469.1 ± 24.5	608.4 ± 80.7	1,497.17	<0.001
TG (mg/dl)	162.3 ± 131.3	172.9 ± 154.0	191.6 ± 155.6	193.2 ± 140.7	2.16	0.091
TC (mg/dl)	189.7 ± 36.5	192.6 ± 38.9	190.6 ± 38.2	202.4 ± 42.6	4.55	0.004
HDL-C (mg/dl)	41.0 ± 10.0	42.6 ± 25.2	38.5 ± 11.4	38.2 ± 10.0	3.83	0.010
LDL-C (mg/dl)	117.3 ± 87.1	115.6 ± 29.4	115.4 ± 26.6	126.6 ± 31.9	2.26	0.080

Values are presented as mean ± SD. P-values are obtained from one-way ANOVA.

BMI, body mass index; HC, hip circumference; HDL, high-density lipoprotein cholesterol; LDL, low-density lipoprotein cholesterol; WC, waist circumference; SUA, serum uric acid; TC, total cholesterol; TG, triglycerides.

**Table 5:** Prevalence of overweight, obesity I, obesity II, and obesity III between the SUA quartiles.

**Variable**	**Prevalence** ***n*****, (%)**				**Total**	***P*** **-values for trend**
	**Overweight**	**Obesity I**	**Obesity II**	**Obesity III**		
Number (*n*)	92	206	205	318	821	
Q1	45 (21.8)	68 (33.0)	56 (27.2)	37 (18.0)	206	<0.001
Q2	26 (12.7)	58 (28.3)	55 (26.8)	66 (32.2)	205	0.043
Q3	17 (8.3)	52 (25.4)	55 (19.0)	81 (39.5)	205	0.314
Q4	4 (2.0)	28 (13.7)	39 (25.0)	134 (65.4)	205	<0.001

The p-value is for trend when the prevalence has compared between the SUA quartiles.

### Multiple linear regression analysis for SUA level

Table 6 shows the relationship between SUA and risk factors of obesity for using multiple linear regression model. Overall, male (β = 69.018, 95% CI: 52.861–85.175, *p* < 0.001) and high TC level (β = 16.228, 95% CI: 6.855–25.602, *p* = 0.001) were significantly more likely to have higher levels in the SUA. Compared with the overweight, obesity I (β = 29.978, 95% CI: 4.173 – 55.783, *p* = 0.023), obesity II (β = 34.854 95% CI: 8.766−60.943, *p* = 0.009), and obesity III (β = 97.107, 95% CI: 71.810 - 122.403, *p* < 0.001) were more likely to have higher levels in the SUA levels. On the contrary, older age (β = −2.222, 95% CI:−2.992 to−1.451, *p* < 0.001) and high HDL level (β = −22.109, 95% CI:−40.652 to−3.566, *p* = 0.020) were significantly more likely to have lower levels in the SUA levels.

**Table 6:** Multiple linear regression between SUA and risk factors of obesity in the model.

**Variables**	**β (95% CI)**	***t***	***P*** **-value**
**Gender**			
Female (Ref.)	-	-	-
Male	69.018 (52.861 to 85.175)	8.385	<0.001
Age (cont.)	−2.222 (-2.992 to−1.451)	−5.658	<0.001
**BMI category**			
Overweight (Ref.)		-	-
Obesity I	29.978 (4.173 - 55.783)	2.280	0.023
Obesity II	34.854 (8.766 - 60.943)	2.622	0.009
Obesity III	97.107 (71.810 - 122.403)	7.535	<0.001
TG (cont.)	0.400 (-4.661 to 5.462)	0.155	0.877
TC (cont.)	16.228 (6.855 to 25.602)	3.398	0.001
HDL-C (cont.)	−22.109 (-40.652 to−3.566)	−2.340	0.020
LDL-C (cont.)	3.598 (-2.903 to 10.100)	1.086	0.278

This model R = 0.536, R-square = 0.287, Adjusted R-square = 0.279.

BMI, body mass index; HDL, high-density lipoprotein cholesterol; LDL, low-density lipoprotein cholesterol; SUA, serum uric acid; TC, total cholesterol; TG, triglycerides.

## Discussion

Obesity has become a health issue in the past decade in China. It has been recognized as a risk factor for various clinical diseases and adverse health outcomes, HUA being one of them (23). However, SUA is not only associated with obesity but also has a significant positive correlation with other clinical diseases such as diabetes, hypertension, and ischemic heart disease. Although these clinical conditions are also associated with obesity, SUA is independently associated with all of these conditions (24). Thus, patients with HUA are more likely to develop diabetes, hypertension, and ischemic heart disease than patients without HUA. Elevated SUA levels also increase morbidity and mortality in these patients (25). The strong correlation between gout, SUA levels, obesity, and metabolic syndrome (26, 27). Notably, most patients with HUA do not have gout. However, elevated SUA levels still increase the risk of gout (28). This study analyzed the prevalence of HUA in obese/overweight adults in China by analyzing the metabolic status and related influencing factors of obese (even severely obese) hospitalized patients and evaluated the relationship between SUA and obesity.

In this present study, the prevalence of HUA was 69.8% (77.0% in men and 65.6% in women), SUA levels are higher in men than in women, and the prevalence of HUA increases with increasing BMI. Similar results were found in previous studies (29, 30). However, the prevalence of the HUA in mainland China was 13.3% (19.4% in men and 7.9% in women), much lower than our results (8). Thus, our findings suggested that the HUA is significantly higher in severely patients with obesity. However, studies have found that HUA is more common in women than men (31). Additionally, in a 10-year follow-up study, BMI increased significantly with SUA levels regardless of race and gender, related to different regions, races, and environmental exposures (32). The protentional factor would be the mechanism that may be related to the effect of estrogen on renal tubular processing of SUA (33). Although gender did not differ significantly in demographic variables, further large-scale epidemiological studies are needed to investigate the effect of gender on HUA.

The correlation analysis shows that SUA is positively correlated with BMI before and after adjusting for age and gender. A positive correlation between BMI and SUA levels was reported in healthy individuals in Jiangsu Province (34), China, in a study consistent with our study. HUA is associated with an increased risk of weight gain, possibly with an accumulation of visceral fat area (35). When subjects were divided into different groups based on BMI levels, SUA levels were elevated in the higher BMI group, especially those with obesity III. In the Q4 high uric acid level group, the number of people increased as obesity increased. It can be seen that the higher the degree of obesity, the higher the uric acid level, and the more likely it is to appear HUA. The degree of obesity is the most important risk factor for HUA. How can this correlation be explained? According to Tsushima reports (36) increased uric acid secretion in adipose tissue in patients with obesity. In patients with obesity, excessive fat accumulation in patients with obesity produces and secretes uric acid, and is associated with HUA of the overproduction type. This may provide a possible mechanism for the relationship between BMI and SUA.

According to a report to Yu Tsushima (36), in patients with obesity, excess fat accumulation in obesity produces and secretes uric acid and is associated with excess production of HUA, which may provide a possible mechanism for the relationship between BMI and SUA. Although a positive correlation between obesity and SUA levels has been reported in previous studies, the mechanism by which uric acid increases in obesity has not been well-elucidated. Many factors can cause HUA, including chronic kidney disease, metabolic syndrome, and dietary habits (37). Interestingly, we found that age was a protective factor against HUA. However, previous studies have shown that HUA increases with age (32). This variability may be due to the small sample size of our study and the differences in diet, ethnicity, lifestyle, and comorbidities in our study population from other studies. We also found that HDL-C is a protective factor against HUA. In previous epidemiological studies, patients with HUA are often associated with dyslipidemia (decreased HDL-c levels and elevated TG levels) (38). The underlying mechanism is that low HDL-c levels increase the risk of kidney damage, possibly leading to decreased uric acid excretion (39). HDL-C and age may be independent predictors of HUA (40). The increased levels of SUA in subjects with obesity may involve two factors: excessive uric acid production and impaired renal excretion. A study in subjects with visceral fat showed that elevated uric acid levels were strongly influenced by hypersecretion of uric acid, with decreased urea excretion and clearance (41). In addition, visceral fat accumulation induces a massive influx of plasma-free fatty acids into living veins and the hepatic portal vein, thereby stimulating triglyceride synthesis, leading to a surge in uric acid production by activating the uric acid synthesis pathway (42).

## Limitations

There are some limitations of our study. First, the cross-sectional nature of the data does not demonstrate a causal relationship between SUA and obesity. Second, the sample size of this study is relatively small, so our findings are not representative of the entire population of China. In addition, our study was in patients above the overweight level and did not enroll in the normal-weight population, which may not apply to other ethnic groups. It has been observed that SUA is a critical determinant of BMI change, and SUA levels can predict subsequent weight gain. However, the underlying mechanism of increased SUA in obese individuals remains to be explored. In the future, more research is needed to determine the mechanisms underlying the association between SUA and obesity in humans.

## Conclusion

In conclusion, our study observed significant associations between SUA and obesity in this decade's cross-sectional study. We mainly showed that a higher SUA level was associated with an increased risk of obesity. The prevalence of HUA in Chinese patients with obesity was ~69.8%. In the BMI categories, the prevalence of HUA was 5.1% in overweight, 15.2% in obesity I, 16.9% in obesity II, and 32.5% in obesity III. In addition, age, and HDL-C, were found to be independent predictors of HUA. Additionally, the increased risk of SUA level was more significant for severe obesity males and young, high TC participants. The serum uric acid levels in Chinese patients with severe obesity declined slightly as age increased. However, compared with the patients with overweight, Chinese patients with obesity I, obesity II, and obesity III were more likely to have higher levels of serum uric acid levels. There is no denying that the relationship between severe obesity and SUA needs more evidence from well-designed studies to confirm our findings in Chinese patients with severe obesity.

## Data availability statement

The raw data supporting the conclusions of this article will be made available by the authors, without undue reservation.

## Ethics statement

The studies involving human participants were reviewed and approved by the Ethics Research Committee of the First Affiliated Hospital of Jinan University. Written informed consent for participation was not required for this study in accordance with the national legislation and the institutional requirements.

## Author contributions

CC and SL: conceptualization and writing-original draft preparation. CC, JR, and WC: methodology, data curation, and formal analysis. CW and ZD: resources, supervision, and project administration. ZD, CC, and SL: writing-review and editing. JR and WC: visualization. All authors contributed to the article and approved the submitted version.

## Conflict of interest

The authors declare that the research was conducted in the absence of any commercial or financial relationships that could be construed as a potential conflict of interest.

## Publisher's note

All claims expressed in this article are solely those of the authors and do not necessarily represent those of their affiliated organizations, or those of the publisher, the editors and the reviewers. Any product that may be evaluated in this article, or claim that may be made by its manufacturer, is not guaranteed or endorsed by the publisher.
